# Supplementary material for: Predicted loss and gain of function mutations in ACO1 are associated with erythropoiesis
Source: Commun Biol. 2020 Apr 23;3:189. doi: 10.1038/s42003-020-0921-5 (PMC7181819; doi:10.1038/s42003-020-0921-5)
Supplement: Supplementary file 1 — Supplementary Information [file 42003_2020_921_MOESM1_ESM.pdf]

Supplementary Tables

Supplementary Table 1. Mean (g/L) and standard deviations of hemoglobin concentration in Iceland and in the UK stratified by gender. N is the number of individuals. Mean is the mean of the mean hemoglobin concentration measurements for each individual. SD: Standard deviation.

	**Iceland**			**The UK**		
	Males	Females	Total	Males	Females	Total
N	139,377	147,245	286,622	183,022	214,466	397,488
Mean	141.4	129.0	135.0	150.0	135.2	142.1
SD	15.7	12.0	15.2	10.1	9.4	12.2

Supplementary Table 2: Heritability (additive model) estimated for hemoglobin concentration using parent-offspring regression and sibling regression, SE: standard error

Phenotype	Heritability, parent-offspring regression (SE)	Heritability, full sibling regression (SE)
Hemoglobin	0.200 [95% CI: 0.194-0.206]	0.293 [95% CI: 0.285-0.300]

Supplementary Table 3. Conditional analysis of the *ACO1* variants in the Icelandic dataset, where each variant is tested with all the other variants as covariates. All coding variant r^2<0.009, between the two common non-coding r^2=0.13. Effect is shown for the minor allele in standard deviations. HGVSp: Human genome variation society nomenclature; LD-class: total number of variants correlating with R2 > 0.8 to the variant (stratified by functional impact class, where HIGH impact variants include stop-gained, frameshift, splice acceptor or donor; MODerate impact variants include missense, splice-region variants and in-frame indels; LOW impact variants include upstream and downstream variants; and LOWEST impact variants include intron and intergenic variants); Amin: Minor allele; Amaj: Major allele

Position (Hg38)	HGVSp	LD class	Amin/ Amaj	P unadjusted	P adjusted	Effect unadjusted	Effect adjusted
chr9:32429450	NP_001265281.1:p.Cys506Ser	1 (0/1/0/0)	A/T	3.0E-24	6.0E-25	-1.61	-1.65
chr9:32418475	NP_001265281.1:p.Thr208Ala	4 (0/1/1/2)	G/A	3.0E-05	1.0E-04	-0.21	-0.19
chr9:32455264	-	2 (0/0/2/0)	C/T	6.0E-07	3.0E-03	-0.023	-0.015
chr9:32450189	NM_001278352.1:c.*78T>C	4 (0/0/1/3)	C/T	1.0E-10	3.0E-05	0.027	0.019
chr9:32418355	NP_001265281.1:p.Arg168Trp	1 (0/1/0/0)	T/C	2.0E-12	1.0E-10	0.21	0.19
chr9:32430494	NP_001265281.1:p.Asn549Ile	2 (0/1/0/1)	T/A	8.0E-06	1.0E-06	0.20	0.22
chr9:32423348	NP_001265281.1:p.Lys334Ter	7 (1/0/1/5)	T/A	5.0E-06	9.0E-06	0.63	0.62

Supplementary Table 4. Comparison of measurements for hemoglobin concentration, iron, and ferritin between carriers of the two rarest *ACO1* variants (Lys334Ter and Cys506Ser) and non-carriers. Percentiles of age of measure (AOM) are shown. Mean: The mean value of measurement for corresponding hematological measurement; SD: Standard Deviation; AOM: Age of measure)

	**Hemoglobin (g/L)**			**Iron (µmol/L)**			**Ferritin (µg/L)**		
**Carrier-status**	**Cys506Ser**	**Lys334Ter**	**Non-carriers**	**Cys506Ser**	**Lys334Ter**	**Non-carriers**	**Cys506Ser**	**Lys334Ter**	**Non-carriers**
**N individuals**	53	63	286,506	27	22	123,640	34	32	172,918
**N meas.**	1,164	1,532	7,329,941	240	93	605,098	339	126	952,427
**Mean N meas.**	25.9	24.3	25.6	8.9	4.2	4.9	10	3.9	5.5
**Mean**	120	145	135	14.4	17	15.8	68.2	146	104
**SD**	12.2	14.3	15.7	4.2	8.3	6.5	55.5	122	93.2
**AOM 25%**	65	57	45	64	53	39	60	40	37
**AOM 50%**	72	68	63	70.5	72	55	70	69	53
**AOM 75%**	78	77	76	79	78	72	79	78	70

Supplementary Table 5. Comparison of hemoglobin concentration, iron, and ferritin levels between different age groups for carriers of the two rarest *ACO1* variants (Lys334Ter and Cys506Ser) and non-carriers. AOM: Age of measure; n: The number of individuals within corresponding age group; mean: The mean value of measurement for corresponding hematological measurement; sd: Standard Deviation.

		**Non-carrier**			**Cys506Ser**			**Lys334Ter**		
**Measure**	**AOM**	**n**	**mean**	**sd**	**n**	**mean**	**sd**	**n**	**mean**	**sd**
**Hemoglobin**	<20	87,414	131	14.6	7	129	12.9	2	149	20.3
	21-40	114,201	137	15.5	19	120	15.1	26	146	17.1
	41-60	107,031	139	13.3	21	121	11.1	33	149	11.6
	61-80	79,314	135	14.4	19	119	9.0	27	143	14.5
	81-100	28,755	125	14.6	6	111	7.5	7	133	11.6
	>100	187	118	14.0	0	-	-	0	-	-
**Iron**	<20	23,076	14.5	6.8	1	11		0	-	-
	21-40	35,126	16.7	6.9	11	12.8	5.5	6	18.9	10.5
	41-60	40,273	16.9	6.3	14	14.8	3.1	11	19.1	8.1
	61-80	33,831	15.7	6.3	10	15.2	2.4	8	13.1	5.4
	81-100	14,075	12.7	5.8	6	17.2	2.8	2	14.6	1.9
	>100	45	9.6	4.5	0	-	-	0	-	-
**Ferritin**	<20	33,522	46	39.2	2	6.5	0.71	1	57	
	21-40	570,60	73	73.2	13	61	60.6	9	118	134.0
	41-60	60,016	114	96.1	17	52	31.8	12	173	149.1
	61-80	44,911	151	102.6	16	74	37.0	10	134	61.8
	81-100	16,284	155	108.4	6	179	161.5	5	179.7	133.2
	>100	47	147	101.8	0	-	-	0	-	-

Supplementary Table 6. Association of variants in *ACO1* with hemoglobin concentration and ferritin. Effect is shown for the minor allele in standard deviations. Significance levels and effects for hemoglobin are shown for the combined analysis. Ferritin measurements were only available from the Icelandic dataset. Amin: Minor allele; Amaj: Major allele; HGVSp: Human genome variation society nomenclature; SD: standard deviation.

					Hemoglobin (N=541,187)		Ferritin† (N=100,054)	
**Position (Hg38)**	**rs name**	**Amin/Amaj**	**MAF Ice/UK (%)**	**HGVSp**	**Effect in SD [95% CI]**	**P**	**Effect in SD [95% CI]**	**P**
chr9:32429450	-	A/T	0.02/-	NP_001265281.1:p.Cys506Ser	-1.61 [-1.92,-1.3]	2.6e-24	-0.77 [-1.1,-0.44]	4.1e-06
chr9:32418355	rs41305321	T/C	0.48/0.12	NP_001265281.1:p.Arg168Trp	0.22 [0.17,0.26]	3.8e-22	0.001 [-0.02,0.02]	0.93
chr9:32450189	rs12985	C/T	35.86/37.08	NM_001278352.1:c.*78T>C	0.02 [0.02,0.03]	4.3e-20	0.001 [-0.01,0.01]	0.83
chr9:32455264	rs7045087	C/T	27.56/30.01	-	-0.02 [-0.02,-0.01]	3.3e-11	0.001 [-0.01,0.01]	0.65
chr9:32418475	rs61753543	G/A	0.16/0.12	NP_001265281.1:p.Thr208Ala	-0.17 [-0.24,-0.11]	2.6e-08	0.04 [-0.08,0.16]	0.51
chr9:32423348	rs745558996	T/A	0.02/-	NP_001265281.1:p.Lys334Ter	0.63 [0.36,0.91]	6.1e-06	0.33 [-0.02,0.68]	0.065
chr9:32430494	rs750337798	T/A	0.21/-	NP_001265281.1:p.Asn549Ile	0.20 [0.11,0.29]	6.9e-06	-0.11 [-0.21,-0.01]	0.024

†Ferritin only available for the Icelandic dataset

Supplementary Table 7 Counts of heterozygotes and homozygotes for the minor alleles of the *ACO1* variants in Iceland and UK datasets. Amin: Minor allele; Amaj: Major allele; MAF: Minor Allele Frequency; Consequence: consequence of sequence variants on transcript or protein level; N het.: number of heterozygous carriers; N homo.: number of homozygous carriers of the corresponding variant.

					Iceland		UK	
**Position (Hg38)**	**rs name**	**Amin/Amaj**	**MAF Ice/UK (%)**	**Consequence**	**N het.**	**N homo.**	**N het.**	**N homo.**
chr9:32429450	-	A/T	0.02/-	Cys506Ser	62	0	-	-
chr9:32450189	rs12985	C/T	35.9/37.1	*78T>C	70,198	19,611	187,883	54,978
chr9:32455264	rs7045087	C/T	27.6/30.0	Intergenic	60,530	11,801	169,281	35,318
chr9:32418355	rs41305321	T/C	0.48/0.12	Arg168Trp	1,458	7	1090	0
chr9:32430494	rs750337798	T/A	0.21/-	Asn549Ile	616	0	-	-
chr9:32418475	rs61753543	G/A	0.16/0.12	Thr208Ala	485	1	653	0
chr9:32423348	rs745558996	T/A	0.02/-	Lys334Ter	67	0	-	-
chr9:32448929	rs147876514	T/C	-/0.01	Arg802Cys	-	-	65	0

Supplementary Table 8. Comparison of genomic evolutionary rate profiling (GERP) site-specific rejected substitutions (RS) scores percentiles for positions over the exome and genome[1].

	GERP score	
**Percentile**	**Exonic positions**	**Genomic positions**
10	-2.6	-2.33
20	-0.083	-0.98
30	1.34	-0.32*
40	2.59	-0.01
50	3.49	0.00
60	4.15	0.17
70	4.65	0.45
80	5.07	1.00
90	5.5	1.95
91	5.54	2.09
92	5.59	2.23
93	5.64	2.4
94	5.68	2.59
95	5.73	2.8
96	5.79	3.07
97	5.85	3.41
98	5.93	3.92
99	6.03	4.71
99.1	6.04	4.82
99.2	6.06	4.94
99.3	6.06	5.07
99.4	6.07	5.21
99.5	6.08	5.34
99.6	6.08	5.48

99.7	6.16	5.61
99.8	6.17	5.75
99.9	6.17	5.9

*For 34%

Supplementary Table 9. Association of variants in *ACO1* with *ACO1* expression based on RNA-sequencing data from 13,163 Icelanders. N is the number of individuals. Effect is shown for the minor allele in standard deviations. Amin: Minor allele; Amaj: Major allele; Consequence: consequence of sequence variants on transcript or protein level; HGVSp: Human genome variation society nomenclature; SD: standard deviation; N Controls: number of controls (non-carriers); N hetero.: number of heterozygous carriers; N homo.: number of homozygous carriers of the corresponding variant.

Position (hg38)	rs name	Amin/ Amaj	Consequence	HGVSp	P val	Effect (SD)	N Controls	N hetero.	N homo.
chr9:32418355	rs41305321	T/C	missense	NP_001265281.1:p.Arg168Trp	0.018	-0.23	13,046	117	0
chr9:32418475	rs61753543	G/A	missense	NP_001265281.1:p.Thr208Ala	0.99	0.0006	13,125	37	1
chr9:32423348	rs745558996	T/A	stop gained	NP_001265281.1:p.Lys334Ter	9.0E-4	-1.04	13,152	11	0
chr9:32429450	.	A/T	missense	NP_001265281.1:p.Cys506Ser	0.46	-0.33	13,158	5	0
chr9:32430494	rs750337798	T/A	missense	NP_001265281.1:p.Asn549Ile	0.49	-0.10	13,111	51	0
chr9:32450189	rs12985	C/T	3'-UTR	NM_001278352.1:c.*78T>C	2.0E-111	-0.29	5,413	6,032	1717
chr9:32455264	rs7045087	C/T	downstream		2.2E-88	-0.28	6,946	5,244	972

Supplementary Table 10. UK Biobank field ID codes for quantitative tested in the current study.

Field ID code	Description
30020	Haemoglobin concentration
30010	Red blood cell (erythrocyte) count
30030	Haematocrit percentage
30000	White blood cell (leukocyte) count
30080	Platelet count
30250	Reticulocyte count
30060	Mean corpuscular haemoglobin concentration
30040	Mean corpuscular volume

Supplementary Figures

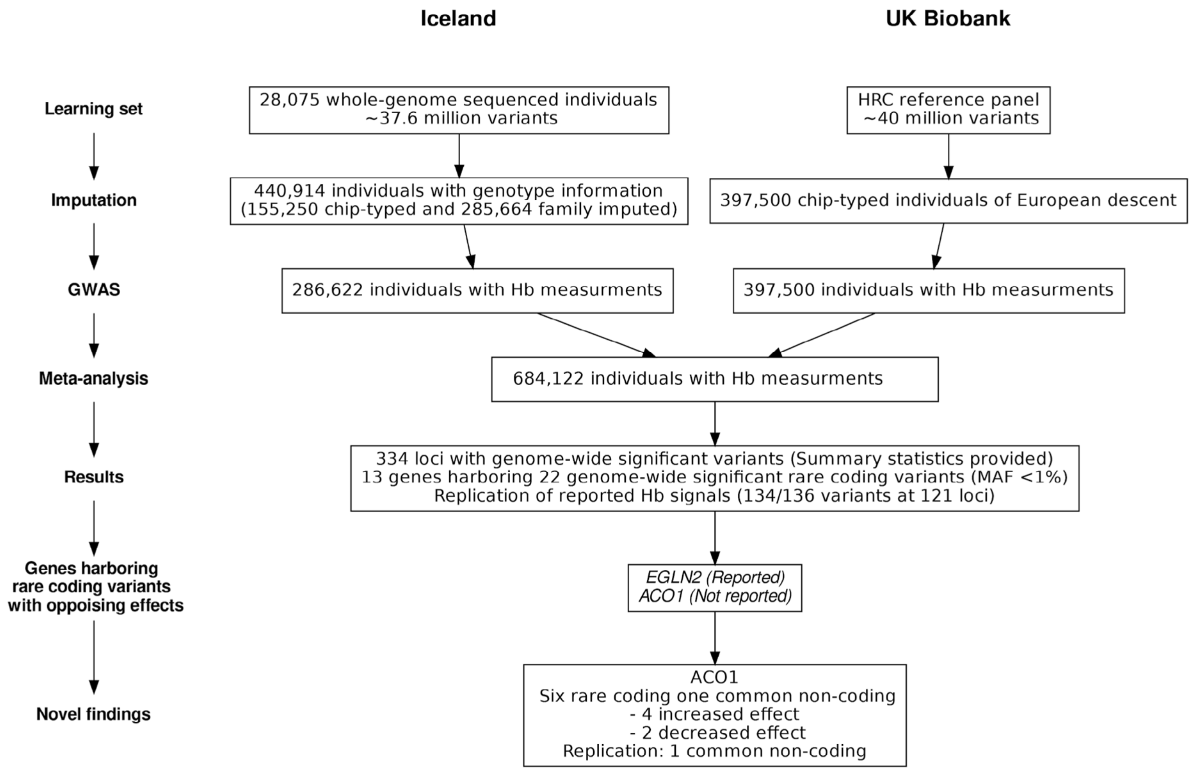

Supplementary Figure 1. A flowchart describing the study design of the hemoglobin concentration meta-analysis of Icelandic and UK Biobank datasets, and a results summary.

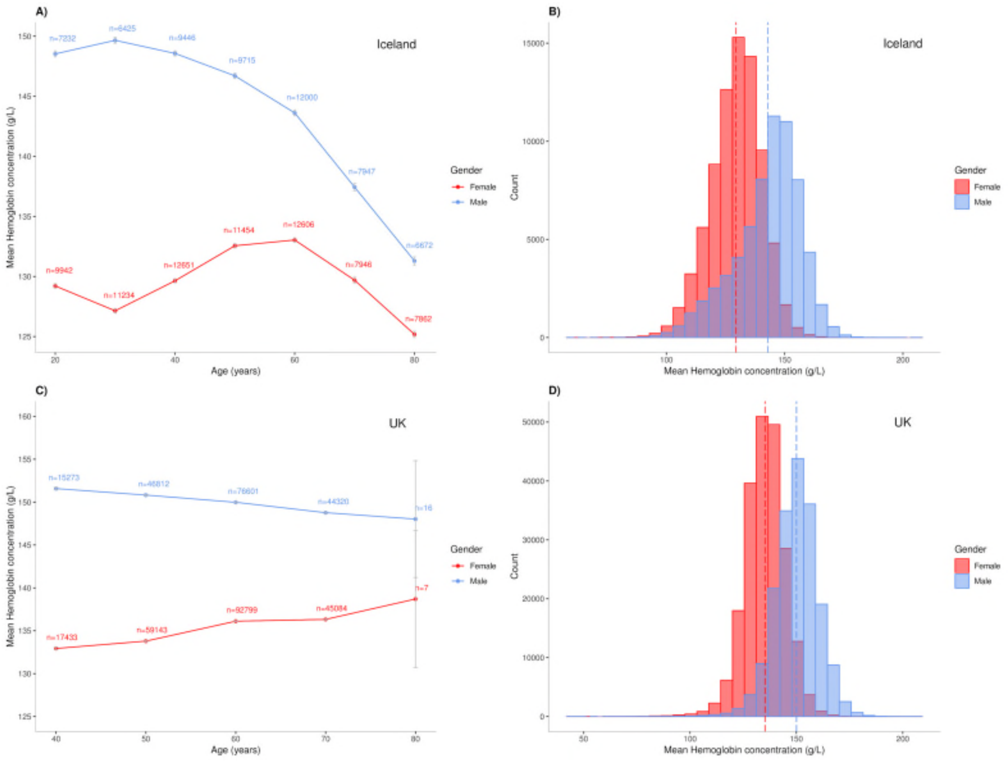

Supplementary Figure 2. Mean Hemoglobin concentration for males and females in Iceland and the UK. A) Mean hemoglobin concentration from 133,132 Icelanders aged 11 to 85 years, divided into seven age groups (number of individuals in each age group shown above each point), and further stratified by sex. Females have lower mean hemoglobin concentration than males at every age group. Hemoglobin concentration decreases with age in men, while a curve of decreased hemoglobin concentration is seen in women at menstrual age. B) Histogram depicting mean hemoglobin concentration of 143,678 individuals in Iceland (64,598 males and 79,080 females). Dashed line shows mean value of mean hemoglobin concentration for each gender (130.3 g/L for females and 143.9 g/L for males). C) Mean hemoglobin concentration from 397,488 individuals from the UK aged 40 to 77 years, divided into five age groups (number of individuals in each age group shown above each point), and further stratified by sex. Females have lower mean hemoglobin concentration than males at every age group. D) Histogram depicting mean hemoglobin concentration of 397,488 individuals in the UK (178,705 males and 218,783 females). Dashed line shows mean value of mean hemoglobin concentration for each gender (135.2 g/L for females and 150.0 g/L for males).

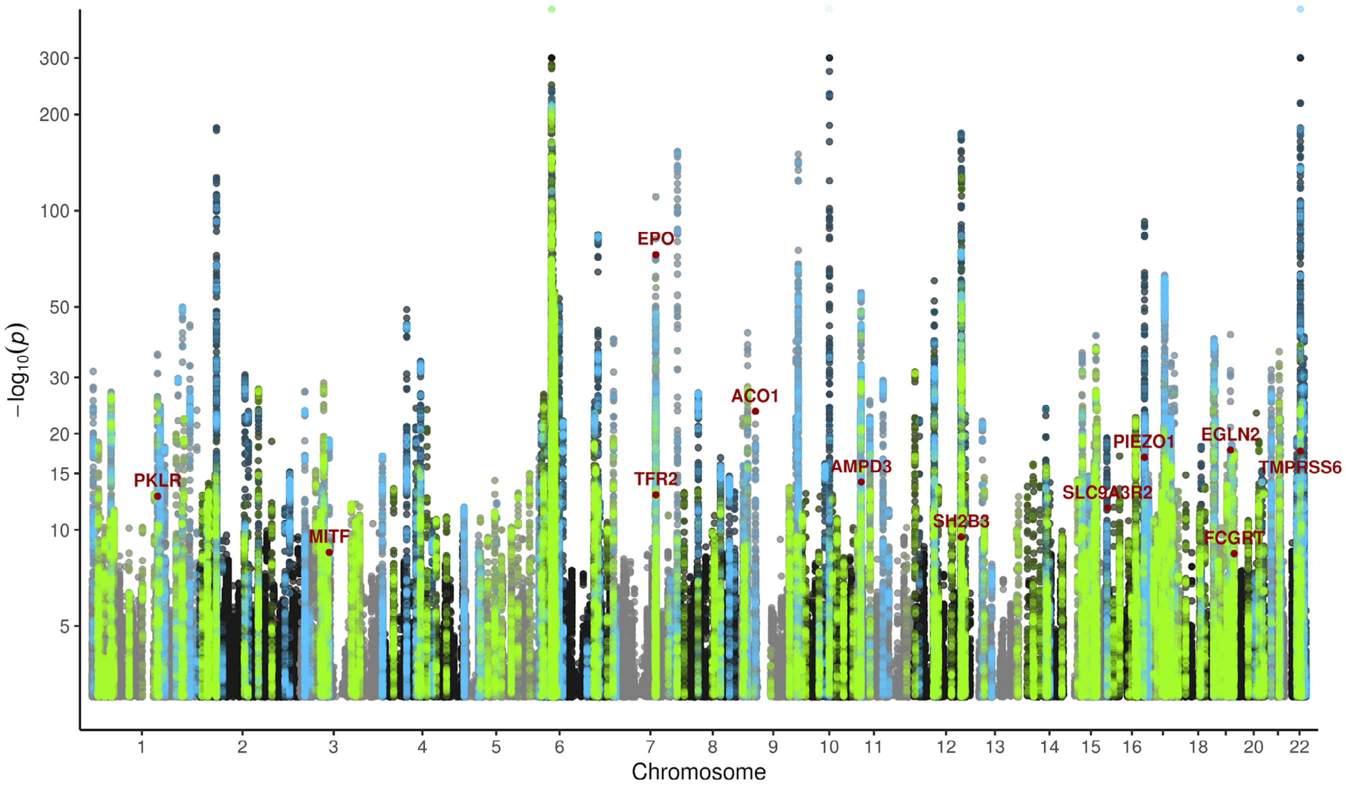

Supplementary Figure 3. Manhattan plot for hemoglobin concentration meta-analysis association results using a combined set of 684,122 individuals from Iceland and the UK. Variants are plotted by chromosomal position (x-axis) and −log10 P-values (y-axis). Blue = loci harboring previously reported hemoglobin concentration associated variants, green = Novel loci harboring genome-wide significant hemoglobin concentration associated variants. The thirteen genes that harbor rare (MAF < 1%) coding sequence variants are labeled in red.

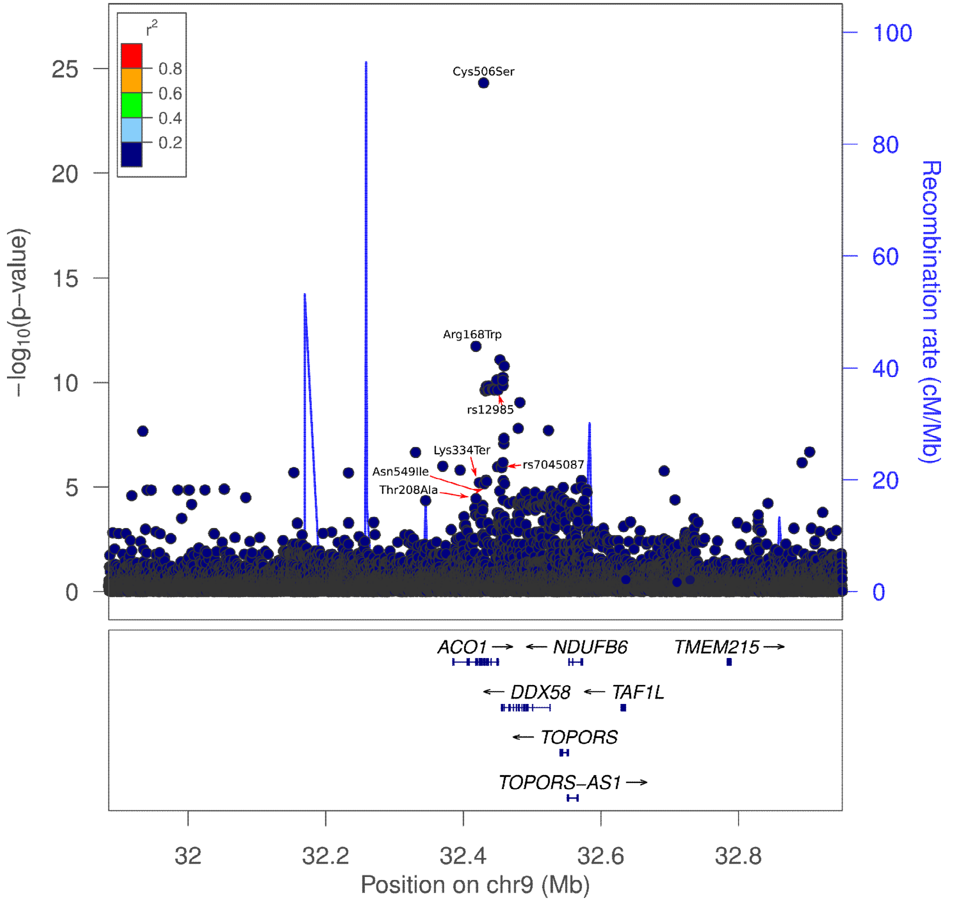

Supplementary Figure 4. Associations of sequence variants at the *ACO1* locus with serum hemoglobin concentration in Iceland. Variants are colored according to correlation (r^2) (legend at top-right). $-\log_{10}P$ values along the left *y* axis and correspond to the variant depicted on the plot. The right *y* axis shows calculated recombination rates at the chromosomal location, plotted as a solid blue line. The seven variants associated with hemoglobin concentration are portrayed using Human Genome Variation Society nomenclature (HGVS) or rs name above the corresponding point or indicated with an arrow. All the five coding markers are distinct signals and none are correlated with the common rs12985[C] or rs7045087[C]. No sequence variant had a more significant corrected P-value than Cys506Ser based on variant annotation.

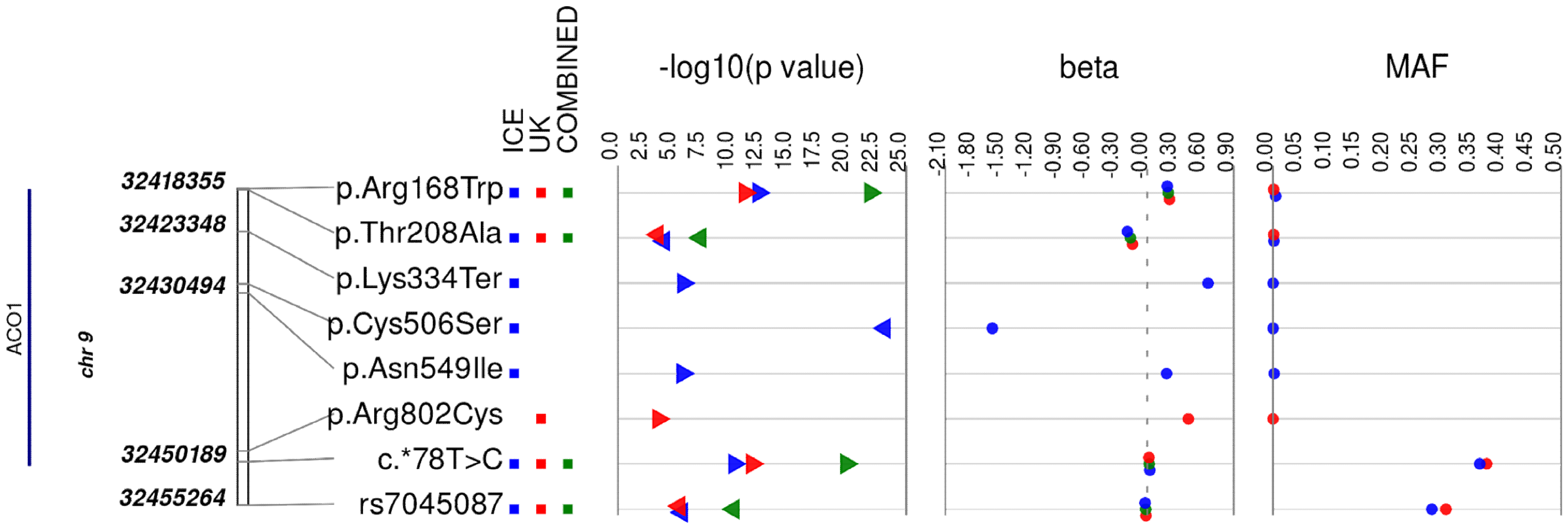

Supplementary Figure 5. Associations of sequence variants at the *ACO1* locus with serum hemoglobin concentration in Iceland and the UK. Variants are colored according to dataset. –log10P values, effect and MAF are for the ACO1 variants is shown along the x axis. The right y axis shows the chromosomal location.

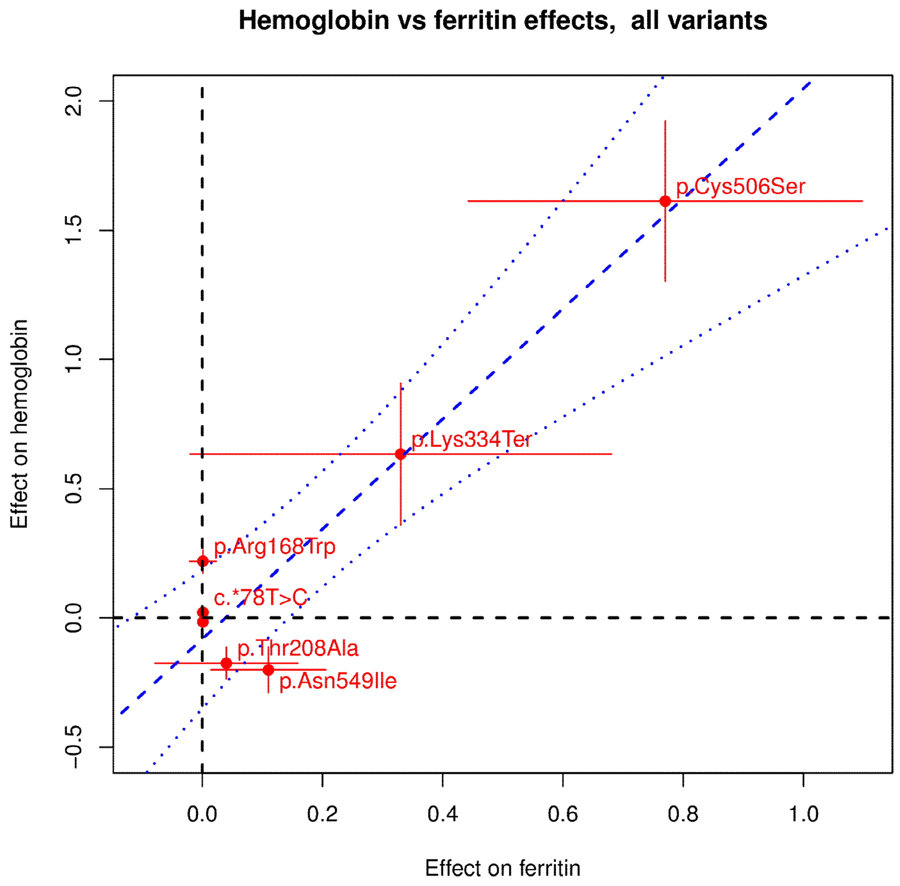

Supplementary figure 6. A scatter plot showing the two common and the five ACO1 rare coding variants Arg168Trp, Thr208Ala, Lys334Ter, Cys506Ser and Asn549Ile. The x-axis shows the effect on ferritin levels in Iceland while the y-axis shows the effect on hemoglobin concentration in the Iceland + UK meta-analysis. For each variant the allele associating with increased ferritin was selected and effect shown in standard deviations (SD). Correlation=0.95 (95%CI: 0.38-1.00), p=0.015

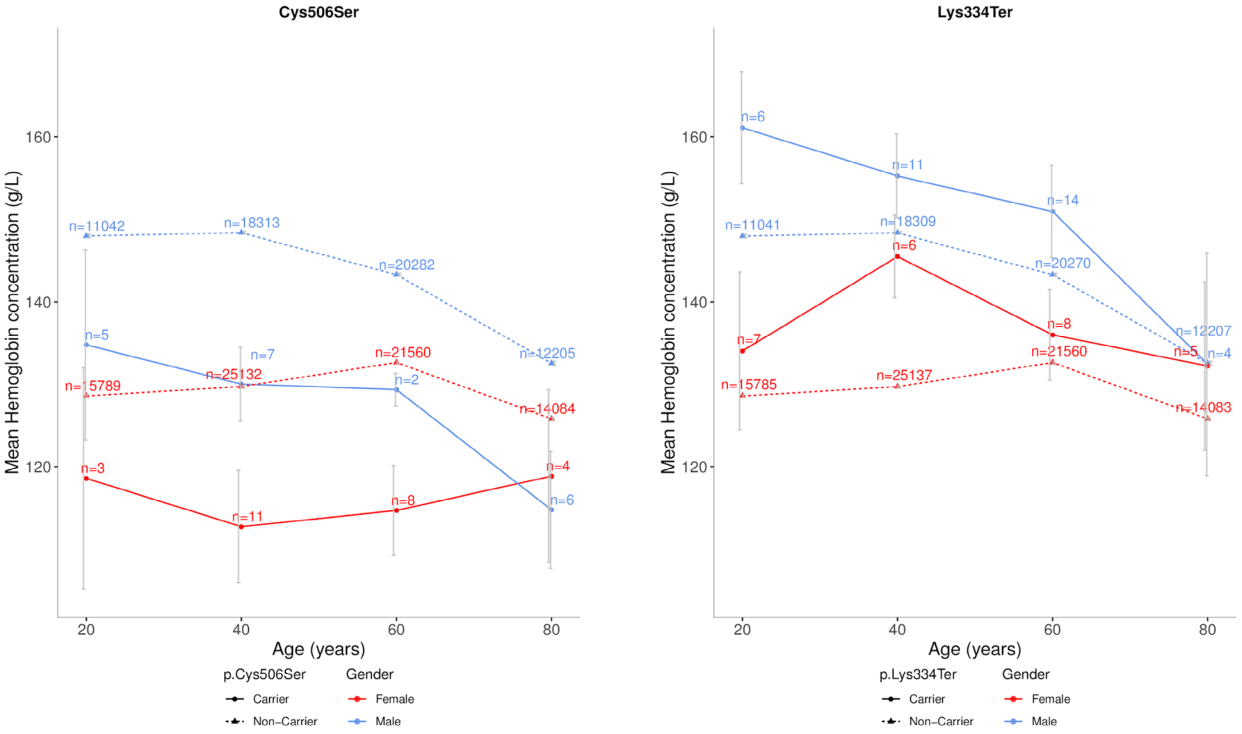

Supplementary Figure 7. A) Mean hemoglobin concentration of 46 male and female carriers of p.Cys506Ser in *ACO1* (n_{males} = 20, n_{female} = 26) in comparison to hemoglobin concentration of non-carriers (n_{total} = 138,453) aged 11 to 85 years old. Cys506Ser associates with decreased mean hemoglobin concentration (Effect = -1.61 SD, P = 2.6 x 10^{-24}). A significant difference in effect is not observed between genders when tested separately (heterogeneity P = 0.59). B) Mean hemoglobin concentration of 61 male and female carriers of p.Lys334Ter in *ACO1* (n_{males} = 34, n_{female} = 28) in comparison to hemoglobin concentration of directly genotyped non-carriers (n_{total} = 138,453) aged 11 to 85 years old. Lys334Ter associates with increased mean hemoglobin concentration compared to non-carriers (Effect = 0.63 SD, P = 6.1 x 10^{-6}) A significant difference in effect is not observed between genders when tested separately (heterogeneity P = 0.57). Number of individuals representing each graphical point are indicated above the point and confidence intervals are depicted as gray vertical lines.

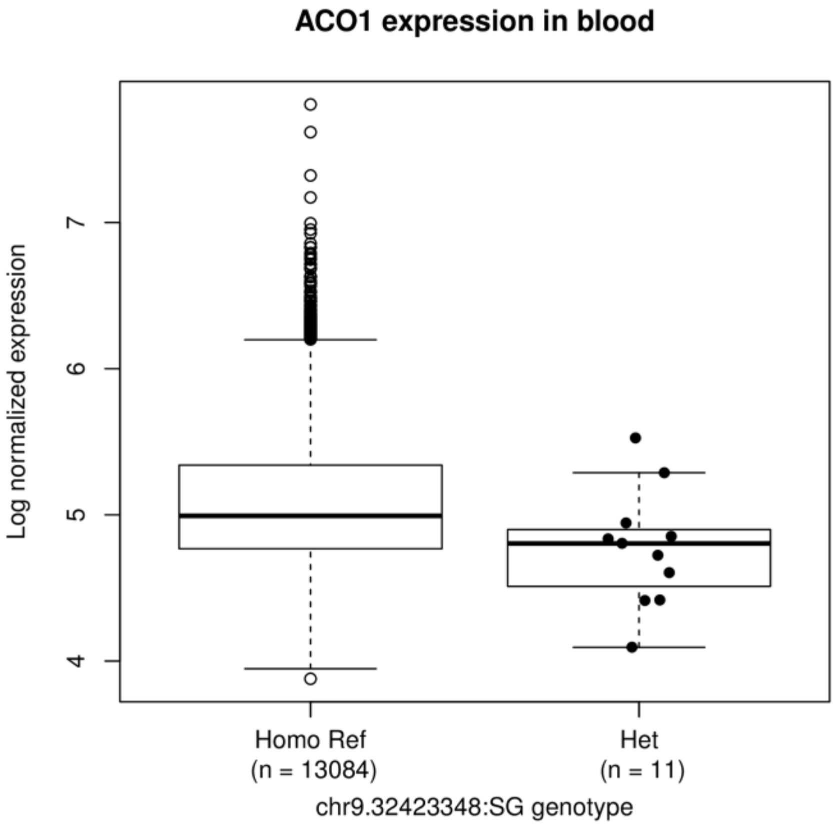

Supplementary Figure 8. Rank-normalized expression levels of *ACO1* mRNA from whole-blood samples of 13,095 Icelanders stratified by genotypes A) rs745558996[T] (Effect = -1.0, P=8.9x10^{-4}). The bottom and top of each box represent the first and third quantiles, the line inside the box is the median and whiskers represent the ±1.5 times the interquartile range

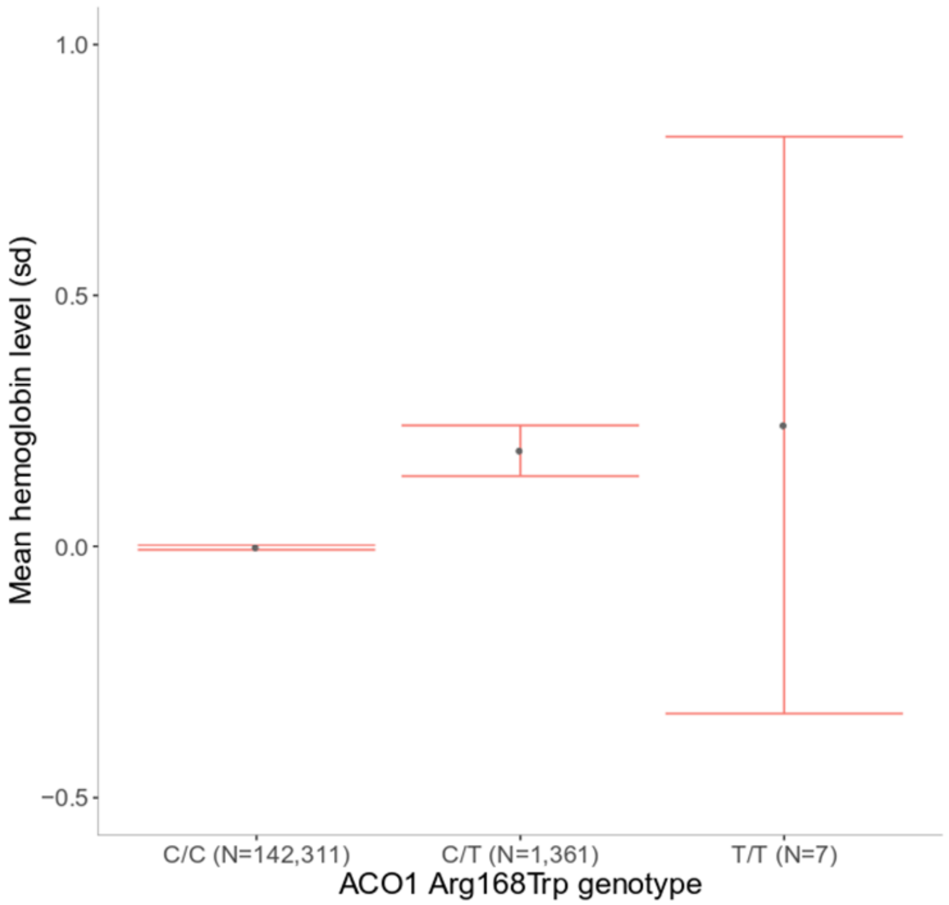

Supplementary figure 9. Genotypic effect of Arg168Trp. Whiskers represent 95% confidence intervals.

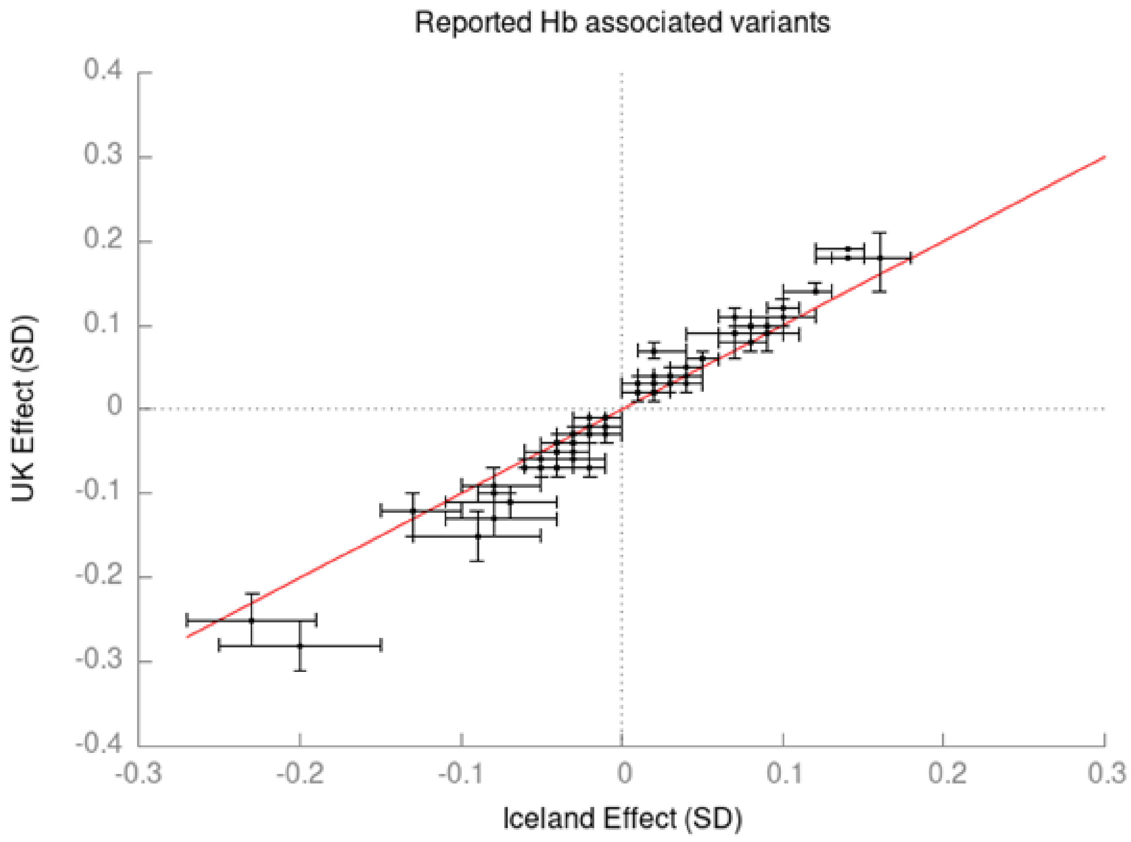

Supplementary Fig. 10. A scatter plot showing hemoglobin concentrations effects (SD) for 131 variants reported to associate with hemoglobin concentration in Europeans for the Icelandic (x-axis) vs. UK datasets (y-axis). There are no significant differences in hemoglobin effect between the datasets

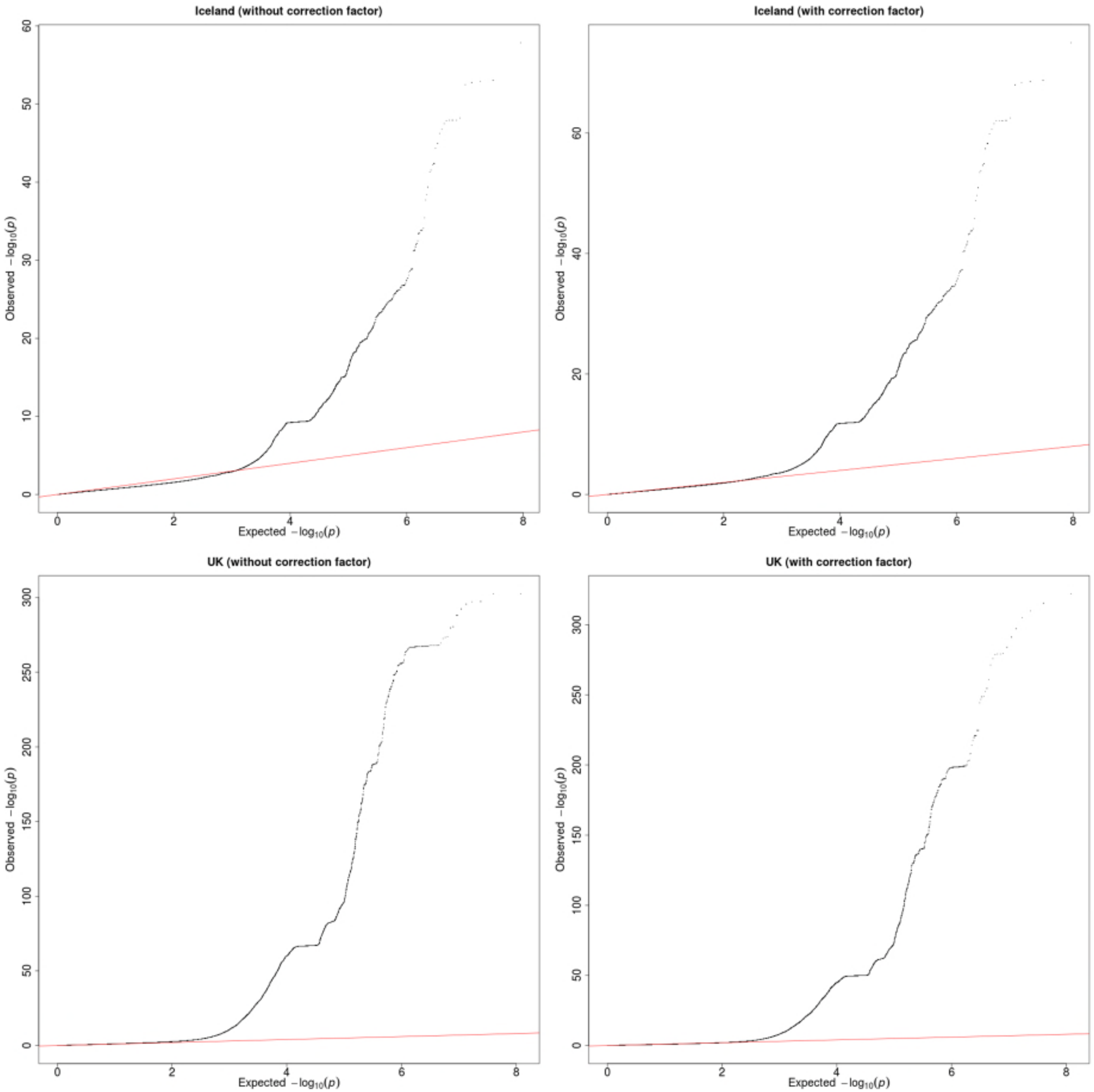
Iceland (without correction factor)
Iceland (with correction factor)
UK (without correction factor)
UK (with correction factor)
Observed $-\log_{10}(p)$
Expected $-\log_{10}(p)$

Supplementary Figure 11. Quantile-quantile plot (QQ-plot) for all sequence variants for the Icelandic and UK datasets included in the meta-analysis of hemoglobin concentration meta-analysis. The estimated correction factor for Hemoglobin concentration based on LD score regression was 0.68 for the additive model in the Icelandic sample and 1.40 in the UK Biobank. The Y axis shows observed −log10 P-values, and the X axis shows the expected −log10 P-values.

1. Davydov, E. V. *et al.* Identifying a high fraction of the human genome to be under selective constraint using GERP++. *PLoS Comput. Biol.* **6**, e1001025 (2010).
