# Supplementary material for: Predicted loss and gain of function mutations in ACO1 are associated with erythropoiesis
Source: Commun Biol. 2020 Apr 23;3:189. doi: 10.1038/s42003-020-0921-5 (PMC7181819; doi:10.1038/s42003-020-0921-5)
Supplement: Supplementary file 3 — Description of Additional Supplementary Files [file 42003_2020_921_MOESM3_ESM.pdf]

Description of Additional Supplementary Files

File name: Supplementary Data

Description: Supplementary data file consists of Supplementary Data 1-9.

Supplementary Data 1: Overview of genome-wide associations with haemoglobin concentration from the meta-analysis of Iceland and UK.

Supplementary Data 2: Associations of variants with hemoglobin concentration in Iceland and the UK for variants previously reported to associate with hemoglobin concentration.

Supplementary Data 3: Rare coding variants (MAF < 1%) reaching genome-wide significant association with hemoglobin concentration in the meta-analysis of the Icelandic and UK datasets.

Supplementary Data 4: Associations of all coding variants in *ACO1* with haemoglobin concentration in the meta-analysis of the Icelandic and UK datasets.

Supplementary Data 5: Sequence variants in *ACO1* associating with haemoglobin concentration in the meta-analysis of the Icelandic and UK datasets.

Supplementary Data 6: Associations of the identified variants in *ACO1* with red blood indices in the meta-analysis of the Icelandic and UK datasets.

Supplementary Data 7: Associations of the identified variants in *ACO1* with anemia phenotypes in the meta-analysis of the Icelandic and UK datasets.

Supplementary Data 8: Associations of the identified variants in *ACO1* with iron metabolism traits in Iceland.

Supplementary data 9: Associations of the top missense variant (Cys506Ser) with 396 disease phenotypes in the Icelandic population.
