# Supplementary material for: Predicted loss and gain of function mutations in ACO1 are associated with erythropoiesis
Source: Commun Biol. 2020 Apr 23;3:189. doi: 10.1038/s42003-020-0921-5 (PMC7181819; doi:10.1038/s42003-020-0921-5)
Supplement: Supplementary file 5 — Reporting Summary [file 42003_2020_921_MOESM5_ESM.pdf]

nature research

Corresponding author(s): Patrick Sulem

Last updated by author(s): Jan 14, 2020

Reporting Summary

Nature Research wishes to improve the reproducibility of the work that we publish. This form provides structure for consistency and transparency in reporting. For further information on Nature Research policies, see Authors & Referees and the Editorial Policy Checklist.

Statistics

For all statistical analyses, confirm that the following items are present in the figure legend, table legend, main text, or Methods section.

n/a	Confirmed	
☐	☒	The exact sample size (*n*) for each experimental group/condition, given as a discrete number and unit of measurement
☐	☒	A statement on whether measurements were taken from distinct samples or whether the same sample was measured repeatedly
☐	☒	The statistical test(s) used AND whether they are one- or two-sided<br>*Only common tests should be described solely by name; describe more complex techniques in the Methods section.*
☐	☒	A description of all covariates tested
☐	☒	A description of any assumptions or corrections, such as tests of normality and adjustment for multiple comparisons
☐	☒	A full description of the statistical parameters including central tendency (e.g. means) or other basic estimates (e.g. regression coefficient) AND variation (e.g. standard deviation) or associated estimates of uncertainty (e.g. confidence intervals)
☐	☒	For null hypothesis testing, the test statistic (e.g. *F*, *t*, *r*) with confidence intervals, effect sizes, degrees of freedom and *P* value noted<br>*Give P values as exact values whenever suitable.*
☒	☐	For Bayesian analysis, information on the choice of priors and Markov chain Monte Carlo settings
☒	☐	For hierarchical and complex designs, identification of the appropriate level for tests and full reporting of outcomes
☐	☒	Estimates of effect sizes (e.g. Cohen's *d*, Pearson's *r*), indicating how they were calculated

Our web collection on statistics for biologists contains articles on many of the points above.

Software and code

Policy information about availability of computer code

Data collection	Not applicable.
Data analysis	We used the following publicly available software for processing of whole-genome sequence data:<br>BWA 0.7.10 mem, https://github.com/lh3/bwa<br>Picard tools 1.117, https://broadinstitute.github.io/picard/<br>SAMtools 1.3, http://samtools.github.io/<br>Bedtools v2.25.0-76-g5e7c696z, https://github.com/arq5x/bedtools2/<br>GraphTyper 1.3, https://github.com/DecodeGenetics/graphtyper<br>Variant Effect Predictor https://github.com/Ensembl/ensembl-vep

For manuscripts utilizing custom algorithms or software that are central to the research but not yet described in published literature, software must be made available to editors/reviewers. We strongly encourage code deposition in a community repository (e.g. GitHub). See the Nature Research guidelines for submitting code & software for further information.

Data

Policy information about availability of data

All manuscripts must include a data availability statement. This statement should provide the following information, where applicable:

- Accession codes, unique identifiers, or web links for publicly available datasets
- A list of figures that have associated raw data
- A description of any restrictions on data availability

Sequence variants passing GATK filters have been deposited in the European Variation Archive, accession number PRJEB15197. RNA-seq data have been deposited in the Gene Expression Omnibus, accession number GSE102870.
The genome-wide association scan summary data will be made available at http://www.decode.com/summarydata

Field-specific reporting

Please select the one below that is the best fit for your research. If you are not sure, read the appropriate sections before making your selection.

☒ Life sciences ☐ Behavioural & social sciences ☐ Ecological, evolutionary & environmental sciences

For a reference copy of the document with all sections, see nature.com/documents/nr-reporting-summary-flat.pdf

Life sciences study design

All studies must disclose on these points even when the disclosure is negative.

Sample size	We had available hemoglobin concentration measurements from 286,622 individuals from Iceland and 397,500 from the UK.
Data exclusions	From the UK Biobank we only used individuals of European ancestry,
Replication	We replicate previously reported associations with hemoglobin concentration.
Randomization	No randomization was used.
Blinding	Not relevant.

Reporting for specific materials, systems and methods

We require information from authors about some types of materials, experimental systems and methods used in many studies. Here, indicate whether each material, system or method listed is relevant to your study. If you are not sure if a list item applies to your research, read the appropriate section before selecting a response.

Materials & experimental systems

n/a	Involved in the study	
☒	☐	Antibodies
☒	☐	Eukaryotic cell lines
☒	☐	Palaeontology
☒	☐	Animals and other organisms
☐	☒	Human research participants
☒	☐	Clinical data

Methods

n/a	Involved in the study	
☒	☐	ChIP-seq
☒	☐	Flow cytometry
☒	☐	MRI-based neuroimaging

Human research participants

Policy information about studies involving human research participants

Population characteristics	The set of 155K chip-genotyped Icelanders used for our study consists of individuals healthy enough to have survived up to the time of recruitment, with the vast majority over 18 years old at the time of sample acquisition.
Recruitment	Subjects in this study were individuals participating in various disease project at deCODE Genetics and had given informed consent.
Ethics oversight	The Icelandic Data Protection Authority and the National Bioethics Committee

Note that full information on the approval of the study protocol must also be provided in the manuscript.
